# Encapsulated Papillary Carcinoma: A Case Report and Review of the Literature

**DOI:** 10.3389/fsurg.2021.743881

**Published:** 2022-02-04

**Authors:** Aikaterini Athanasiou, Fathi Khomsi, Bouquet de Joliniere, Anis Feki

**Affiliations:** Department of Obstetrics and Gynecology, Fribourg Cantonal Hospital Chemin des Pensionnats, Villars-sur-Glâne, Switzerland

**Keywords:** breast cancer, encapsulated breast cancer, fibrovascular cores, sentinel lymph node, biopsy, receptors

## Abstract

Papillary neoplasms are a distinct assemblage of breast lesions whose main characteristic is the presence of fibrovascular cores which are surrounded by epithelial cells. Papillary lesions are of heterogenous nature, with similar clinical behavior and histomorphologic characteristics. Their biological patterns, however, can be quite different. According to the World Health Organization (WHO) (2019), breast tumors have been recently classified into five subdivisions of papillary neoplasms. They are namely: intraductal papilloma, papillary ductal carcinoma *in situ*, encapsulated papillary carcinoma (EPC), solid-papillary carcinoma and invasive papillary carcinoma. Despite the papillary architecture being easily recognized, histological variations are diagnostically challenging. The presence or absence of myoepithelial cells in the papillary cores can distinguish the malignant from the benign lesions respectively. EPC is a rare, histologically unique carcinoma type whose main characteristic is a thick fibrous capsule at the periphery and a prolific cell structure with fibrovascular stalk support. A characteristic feature is the absence of myoepithelial cells at the surrounding thick fibrous capsule. Usually, EPC maintains a slowly developing tumor despite the absence of myoepithelial cells. An EPC case presents diagnostic difficulties since it bears close resemblance to malignant and benign papillary breast lesions. Upon a clinical and radiological evaluation, EPC commonly appears as a benign lump. In mammography, the tumor is frequently found in a retroareolar position as a well-defined mass. On the other hand, in an ultrasound, the tumor will appear as a cystic lesion characterized by solid components. The clinical picture of EPC is usually an asymptomatic benign mass which at times can be felt through auto-palpation or screening mammography. A bloody nipple discharge is regarded as a common symptom. We report a case of an EPC of a 81-year-old woman who presented with a mass in the left breast.

## Case Presentation

An 81-year-old female patient (4G-3P, 158 cm, 65 kg) presented with a large lump in the left breast for 1 year which was increasing in size gradually. The patient had no history of malignancy or family history of breast cancer. Physical examination found a 3.0 cm well-defined and freely mobile mass situated in the left inner inferior quadrant. No axillary nodes were palpable. Routine laboratory investigations were within normal limits.

Ultrasonography and mammography showed a 2.9 × 1.6 × 2.7 cm lump in the left inner inferior quadrant, which was categorized as Breast Imaging-Reporting and Data System (BIRADS) 0, without lymph node involvement.

Core needle biopsy revealed a papillary tumor of low grade with 100% estrogen receptor positivity and 98% progesterone receptor positivity, and Ki-67 proliferation rate was between 15 and 20%.

The patient underwent a left total mastectomy as per her wish, with excision of two axillary sentinel lymph nodes and one accessory lymph node, as it was decided with the multidisciplinary tumor board of the hospital.

Final histology concluded to an EPC without signs of invasiveness. The lymph nodes were negative.

Regarding the follow-up of the patient, a history and physical examination was planned 2 times per year for 5 years and after that, every 12 months. Mammography was scheduled to be done annually. An active lifestyle, a balanced diet, limited alcohol intake, and maintaining a healthy weight were all recommended to the patient.

### Epidemiology

Encapsulated papillary carcinoma (EPC) is considered rare breast cancer since it refers to only 1–2% in women (1). Additionally, the prognosis is excellent in the absence of invasiveness (2). The commonest population group regarding ethnicity, are Caucasian women from 55 to 67 years old that are in the post-menopausal stage (3, 4). It is usually diagnosed at an average age of 67 years (5, 6). EPCs with invasion appear at an average age of 59.3 years, which is considered relatively young (1). Male patients cover about 2–7% of EPC cases (1, 7).

### Pathologic Features

**Figure 1:**
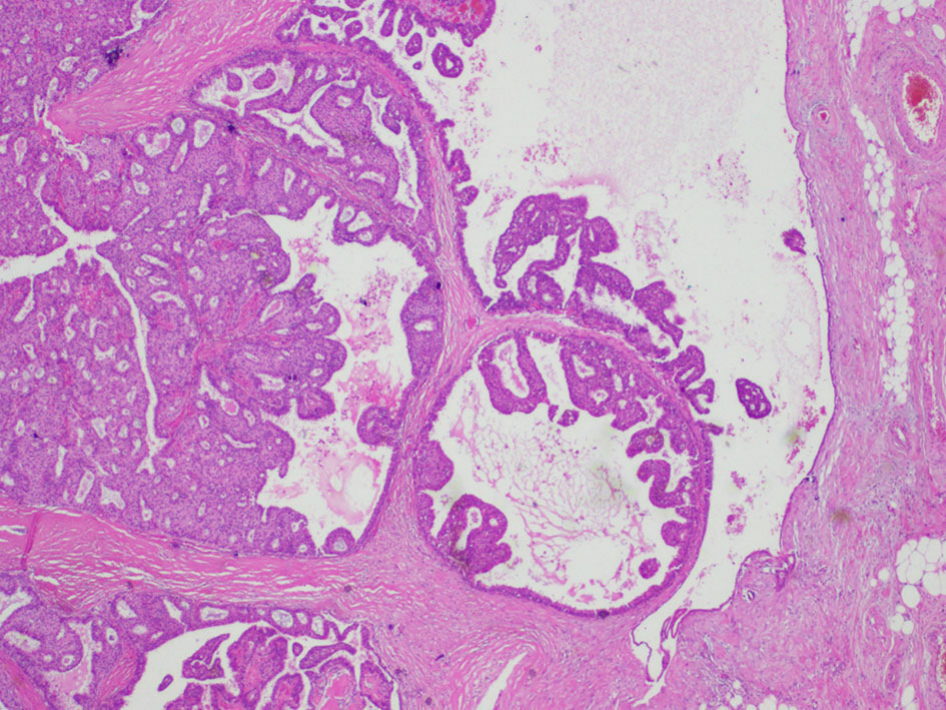
Histological findings of encapsulated papillary carcinoma (EPC). The tumor nodule is surrounded by a thick fibrous capsule.

**Figure 2:**
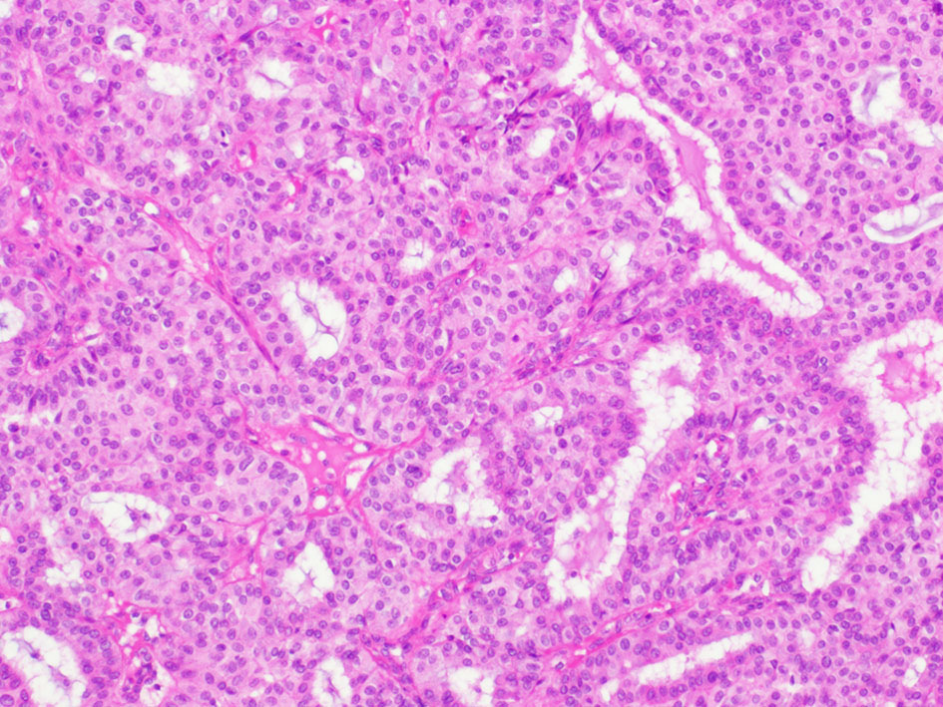
Histological findings of encapsulated papillary carcinoma (EPC). Papillary proliferation inside the dilated ducts.

Upon histological observation, EPC seems like a sole lesion within a cyst (8). Morphologically, the existing papillary architecture is a mesh where branches of fibrovascular cores are lined with neoplastic epithelial cells (8). Peripherally, EPC is characterized by a capsule composed of fibers of varying thicknesses (8) (Figure 1). Inside this capsule, luminal epithelial cells proliferate in combination with thin fibrovascular cores (Figure 2). A myoepithelial layer is absent both in papillary structures and in the capsule (9).

Sometimes, ductal carcinoma *in situ* (DCIS) and/or invasive ductal carcinoma can be associated with EPC (10). When the stroma is infiltrated by abnormal neoplastic cells crossing the fibrous capsule, invasion is present (8). DCIS in the nearby breast tissue has been recorded in 28.6–70% of cases (1, 8).

There are characteristics, however, which justify the noninvasive character of the tumor, such as very good prognosis, very small number of metastasis incidents, limited lymphovascular invasion, and relapse (11). Nevertheless, there are some cases of invasive EPC that cross out of the fibrous capsule and show lymph node (LN) metastasis, such as EPC with invasion (11). In addition, these EPCs are devoid of myoepithelial cells around the capsule, indicating characteristics of invasive ductal carcinoma. This causes a diagnostic challenge regarding invasion (11).

In the scientific community, it is widely believed that EPC is a DCIS variation, as it exhibits the following characteristics. Initially, there is a fibrous capsule, there is no stromal reaction, and there is a slowly progressing clinical pattern (12). Conversely, there is another view supporting that a tumor is a slowly-developing form of invasive carcinoma with expansive potential (12). This view is justified by the lack of myoepithelial cells that do not support the idea of an *in situ* carcinoma (12). Moreover, the possibility of finding LN metastasis supports the latter opinion further (12).

The medical opinion that prevails is to classify it as DCIS (1).

The usual classification of EPC is a noninvasive type of breast cancer at a rate of about 60% (1). Additionally, almost 40% of the cases are classified as a varied subgroup of low-grade DCIS (1). As to the EPC classification of one of the two groups, opinion is still divided.

The WHO Classification of Tumors of the Breast (2012) defines EPC as with or without invasion. Due to the slow-growing nature of EPC, WHO suggests considering it like a DCIS.

Having examined the overall molecular modifications, it was implied that an EPC shares the characteristic features of DCIS to a greater extent than those of invasive carcinoma (12).

At times, EPC has been linked with cancer due to the presence of invasive ductal carcinoma on the condition that an invasive component is present outside the fibrous capsule (9). In fact, biological characteristics of both DCIS and invasive carcinoma are present, with the latter being predominant (13). This case is defined as EPC with invasion. On rare occasions, both EPC and EPC with invasion can result in axillary metastasis. Concerning receptors, EPC retains a diffusing pattern of hormonal receptors. Progesterone and estrogen receptors (PRs, ERs) are categorized as positive, while human epidermal growth factor receptor-2 neu (HER2neu) is classified as negative in most of the cases (1, 8, 9). In 2018, a clinicopathologic study on 49 cases of patients with EPC and an average diagnostic age of 68.5 years performed by Li et al. revealed that 95.9% of tumors were ER- and PR- positive and that only 8 cases exhibited HER/2 1+ immunoreactivity (13).

### Diagnosis

#### Gross Examination

**Figure 3:**
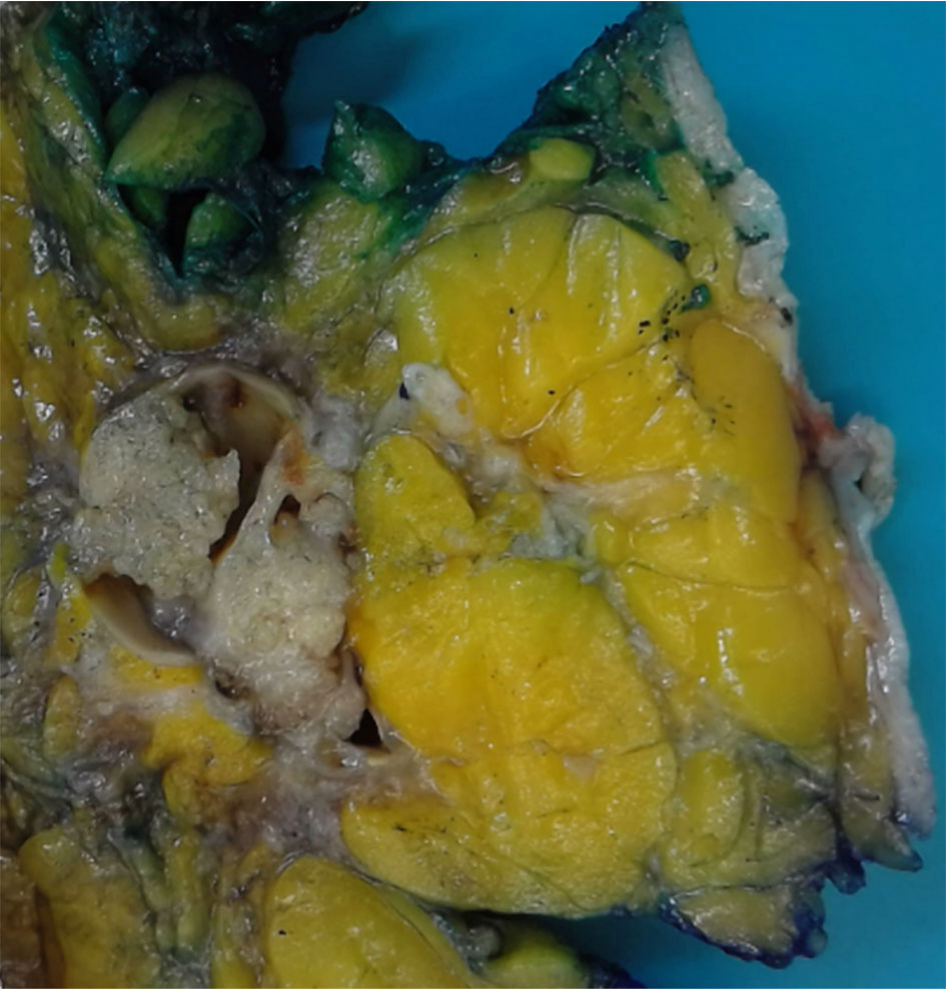
Gross pathology of the specimen shows solid mural nodules and cystic spaces surrounded by a fibrous capsule.

Encapsulated papillary carcinoma (EPC) is usually a large-size tumor (mean: 2 cm) within a large cystic duct (1). Clinically, EPC manifests as a painless lump in the breast, which could be present for several years (1). A common symptom is a bloody nipple discharge, or it can often be asymptomatic and located by screening mammography (1). On gross examination, it seems as a tan white, well-delimited, and intracystic friable tumor (Figure 3).

#### MMG (Mammogram)

In mammographic evaluation, EPC appears as a well-defined, non-calcified, and dense mass with an oval or circular shape (1, 10). If an invasive carcinoma exists, there is a likelihood that in about 50% of cases, mass margins are not so distinct in invaded areas, which are characterized by spiculated margins (14, 15). Regarding calcifications, their existence is not so common, with only 13% of cases reporting them (10).

#### US (Ultrasound)

In the US, EPC presents as a solid mass or as a heterogeneous tumor containing cystic and solid parts (1, 10). Margins are often well-delimited but at times the mass is loosely bordered or surrounded by multi-circular contours, which may indicate malignancy (1, 14). Internal echoes are caused by septations in the cystic part (1). The existence or not of vascularity is defined through a Doppler (16, 17).

#### MRI

Regarding MRI, there is no specificity in the case of EPC (1). An enhancing complex cyst and a multicystic lesion with a solid central component are two of the main EPC characteristics on MRI (18). Sometimes the differentiation of breast malignancies from benign breast lesions can be quite challenging by using conventional imaging modalities. MRI plays an important role in distinguishing such lesions (19).

### Core Needle Biopsy

Identification of histologic characteristics of these tumors on core needle biopsy is critically important (20). Performing core needle biopsy can indicate the nature of papillary lesions as benign or malignant, but it cannot distinguish between invasiveness and non-invasiveness of a tumor (4). In order to define the tumor morphologically, nearby *in situ* and invasive diseases may be located peripherally in EPC, and they are likely to escape sampling in core needle biopsy. To make diagnosis more efficient, it is essential to include the mass wall where a lack of myoepithelial markers will be observed (10).

A physician has the option of surgery to excise such lesions without performing a biopsy pre-operatively (1). However, a biopsy is obligatory post-operatively in papillary lesions to expose the risk of peripheral lesion-malignancy (1). This diagnostic path is challenging, and only the existence of resection specimens can provide a definitive answer (20).

### Management

Controversy still clouds therapeutic recommendations for EPC (1). When an actual invasion is not present, EPC is evaluated, classified, and managed as an *in situ* disease (8). On the other hand, when an invasion is present, classification and management are decided according to invasive features (8). The common treatment choice is complete surgical resection (1, 7). An investigative adjunct can be sentinel lymph node biopsy, which is recommended due to minimal LN metastasis (7, 8). Pure EPC cases, cases with DCIS association, and cases with invasion are all possible candidates for recurrence (7).

According to specific selective criteria, hormonal therapy, chemotherapy, and radiotherapy may ensue (21, 22). Specifically, adjuvant radiotherapy is a treatment option in cases where DCIS and/or invasion is involved. Additionally, if invasive tumors are histologically aggressive, the treatment choice is adjuvant chemotherapy (1, 8). Finally, adjuvant hormonal therapy is an option for patients who cannot undergo surgery, patients whose tumors appear repeatedly, or patients whose age is lower than 50 years old (7, 8, 22).

### Prognosis

The clinical path of EPC has an excellent prognosis. It is recommended that meticulous investigation should be performed regarding high nuclear grade and invasion, as the stage of the tumor and selected treatment will be decided based on these elements (8). On the whole, the behavior of EPC is very good, with scarce local relapse, few distant metastases, or death owing to breast cancer (8). Specifically, this good prognosis results from the slow-growth nature of the tumor, with 10-year survival reaching about 100% and 10-year disease-free survival reaching 91% (1, 8).

## Data Availability Statement

The original contributions presented in the study are included in the article/supplementary material, further inquiries can be directed to the corresponding author/s.

## Ethics Statement

Written informed consent was obtained from the individual(s) for the publication of any potentially identifiable images or data included in this article.

## Author Contributions

All authors listed have made a substantial, direct, and intellectual contribution to the work and approved it for publication.

## Conflict of Interest

The authors declare that the research was conducted in the absence of any commercial or financial relationships that could be construed as a potential conflict of interest.

## Publisher's Note

All claims expressed in this article are solely those of the authors and do not necessarily represent those of their affiliated organizations, or those of the publisher, the editors and the reviewers. Any product that may be evaluated in this article, or claim that may be made by its manufacturer, is not guaranteed or endorsed by the publisher.
